# Reliability of Actigraphy for the Assessment of Sleep and Circadian Rhythms in Rett and Related Syndromes

**DOI:** 10.1111/jir.70068

**Published:** 2025-12-04

**Authors:** Breanne Byiers, Alyssa Merbler, Elijah Lockhart, Chantel Burkitt, Frank Symons

**Affiliations:** ^1^ Department of Educational Psychology University of Minnesota Minneapolis Minnesota USA; ^2^ Gillette Children's St. Paul Minnesota USA

**Keywords:** actigraphy, intellectual and developmental disabilities, reliability, Rett syndrome

## Abstract

**Purpose:**

Actigraphy is being increasingly used to assess sleep and circadian rhythms among populations with intellectual and developmental disabilities and genetic syndromes, including Rett syndrome and related disorders, but the reliability of these measures in these populations is unclear. The primary purpose of the current study was to evaluate the impact of recording duration on the reliability of various measures of sleep and circadian rhythm in Rett and related syndromes.

**Method:**

Two 14‐day recordings were collected between 4 and 12 weeks apart in a sample of 30 individuals (aged 2–36 years; 97% female). Reliability was estimated by calculating statistics based on 3, 5, 7, 10 or 13–14 nights of recording.

**Results:**

Most measures of average sleep quality could be reliably estimated with 7–10 nights. Measures of night‐to‐night variability in sleep timing showed poor reliability at all recording durations, whereas night‐to‐night variability in sleep duration showed adequate reliability at 5–7 days of recording. The reliability of measures of circadian rhythm was highly variable.

**Conclusions:**

The results suggest that the optimal recording durations for actigraphy in this population vary based on the specific metrics of interest, but most can be measured reliably.

Individuals with intellectual and developmental disorders (IDDs), particularly those with developmental and epileptic encephalopathies such as Rett syndrome, experience more sleep problems, including increased sleep fragmentation and insomnia, and are more prone to disruptions in sleep–wake circadian rhythms than their typically developing peers (Maaskant et al. 2013; Proost et al. 2022; Surtees et al. 2018). Rett syndrome, a genetic syndrome affecting almost exclusively females and caused by loss‐of‐function mutations of the methyl‐CPG binding protein 2 (MECP2) gene (Amir et al. 1999; Neul et al. 2010; Gold et al. 2024), is one specific form of IDD in which poor sleep is a common problem (Boban et al. 2018; Nomura 2005; Wong et al. 2015; Young et al. 2007; Veatch et al. 2021). This extends to atypical forms associated with mutations in other genes, including the cyclin‐dependent kinase‐like 5 (CDKL5) and the forkhead box G1 (FOXG1) genes (Percy et al. 2023). Although each of these disorders has a distinct phenotype, they all share several important features, including severe deficits in fine and gross motor function, communication impairments, intellectual disability and a higher prevalence of sleep disorders, particularly frequent night wakings and daytime somnolence (Hagebeuk et al. 2023; Spruyt 2022; Vegas et al. 2018). According to ClinicalTrials.gov, several ongoing or recently completed clinical trials in Rett syndrome include assessments of sleep among their outcome measures, but most of these assessments rely on caregiver reports of sleep, rather than objective measures, suggesting a need for evidence supporting the utility of objective measures of sleep in this population.

Sleep difficulties can have profound impacts on quality of life for affected individuals and their caregivers (e.g., Reddihough et al. 2021; Boban et al. 2018; Coleton and Altevogt 2006). Specific to populations with IDD, there is evidence that sleep problems are associated with increased risk for mood disorders (e.g., Rzepecka et al. 2011; Whitney et al. 2019), worse pain outcomes, increased sedentary behaviour (e.g., Wang et al. 2018), cognitive dysfunction (e.g., inattention, learning), reduced social behaviour and mental health and challenging behaviour problems (e.g., self‐injury; Didden et al. 2002; reviewed in Harper et al. 2023). Circadian rhythms, a related but separate aspect of health and function, regulate the pattern of biological and behavioural processes, such as sleep–wake cycles and hormone secretion patterns. Circadian rhythm disruption is also associated with several neurodevelopmental disorders and can have negative consequences on the immune system, energy levels, cognition and learning and mood disorders (reviewed in Logan and McClung 2019, and Walker et al. 2020).

Actigraphy, which involves using an accelerometer typically placed on the wrist or ankle to measure movement over time, can be used as an objective measure of sleep quality, quantity and circadian rhythms, and is widely used in both clinical and research settings. Although there is a great deal of evidence supporting the reliability and validity of actigraphy to measure sleep and circadian rhythms in typically developing populations (e.g., Smith et al. 2018), there are several reasons to wonder whether the reliability estimates of actigraphy‐derived variables obtained in other populations apply to individuals with Rett and related syndromes, including high rates of sedentary behaviour due to motor disability (Quante et al. 2015; Stahlhut et al. 2019), increased rates of insomnia (Someren 2007; Tascini et al. 2022) and frequency of missing caregiver report data on sleep and wake times (Byiers et al. 2025). Although little work has been done to evaluate the reliability of actigraphy in populations with IDD, in one small study, Laakso et al. (2004) reported poorer agreement between actigraphy and polysomnography in detecting sleep and wake for individuals with motor impairments and IDD relative to those without disabilities.

Estimating reliability is a critical step in establishing the utility of a measure for clinical and research contexts, and a prerequisite to establishing validity. This is particularly important in the context of intervention studies designed to measure within‐individual change, as the reliability of the change score, and therefore the sensitivity of a measure to detect change, is directly proportional to the reliability of the measure at each point in time (Streiner et al. 2015). This is further accentuated in the context of rare diseases given that statistical power decreases as measurement error increases, resulting in the need for larger sample sizes when unreliable measures are used (Williams and Zimmerman 1989). Because actigraphy allows for multiday recordings, the duration of the recording can be increased to allow for averaging across more nights to improve reliability to reach a standard of 0.70 or higher desired for clinical trial outcome measures, when necessary, but longer recording durations increase participant burden and the likelihood of data loss.

To our knowledge, no studies have specifically examined parameters of circadian rhythm based on actigraphy in Rett or related syndromes, although there is some preclinical evidence that circadian rhythms may be disrupted (Li et al. 2015) and thus may be of interest as potential outcome measures in future studies.

The use of actigraphy to measure sleep among individuals with Rett syndrome has been reported previously, with evidence of feasibility (Merbler et al. 2018), and sensitivity to treatment effects (McArthur and Budden 1998). No studies of the reliability of actigraphy‐derived measures of sleep quality, variability, or circadian rhythm variables in Rett or related syndromes have been reported to date. Therefore, the primary purpose of the current study was to identify the number of nights necessary to achieve test–retest reliability of at least 0.70 for several actigraphic metrics of nighttime sleep quality, duration, variability and circadian rhythm across two recordings collected between 4 and 12 weeks apart in a sample of individuals with Rett and related syndromes. This was done using a novel hierarchy of decision‐making rules for data cleaning independent of sleep diaries (diary agnostic), due to the limitations of caregiver report mentioned above (Byiers et al. 2025). A secondary purpose was to evaluate the reliability of bed and rise times for cleaning actigraphy data in this population using this diary‐agnostic approach.

## 1 Methods

### 1.1 Participant Recruitment

All study procedures were approved by the local institutional review board, and informed consent was provided by all legal guardians. Participating individuals and their caregivers were recruited through a local Rett syndrome clinic and regional and national Rett syndrome family support organisations as part of a larger study examining the reliability and validity of potential outcome measures for this population (see Byiers et al. 2023, 2025). To be included in the current analyses, participants had to provide two valid actigraphy recordings of at least 7 days in duration separated by 4–12 weeks and have either a clinical diagnosis of classic or atypical Rett syndrome (with or without genetic testing), or a genetically confirmed diagnosis of a Rett‐like or MECP2‐related disorder, including MEPC2 duplication syndrome, CDKL5 deficiency disorder, or FOXG1 syndrome.

### 1.2 Devices and Measures

Philips Actiwatch 2 (Philips Respironics, Bend, Oregon) devices were used to collect sleep data. At the onset of the study, the Actiwatch 2 was the smallest research‐grade actigraph available to best fit our participants' wrists, commonly used in the paediatric sleep literature (Meltzer et al. 2012), and had preliminary feasibility evidence in Rett syndrome (Merbler et al. 2018). For each recording period, the device was mailed to participants' homes, along with an instruction booklet and paper sleep diary. Caregivers were instructed to place the device snugly on the wrist of the participants' nondominant arm. If the individual did not clearly have a dominant arm, the device was placed on the left wrist. For participants under the age of 36 months (*n* = 3) or for whom the device would not fit snugly on the wrist, the device was placed on the left ankle (Montgomery‐Downs and Tikotzky 2021).

Actigraphy data were analysed using Philips Respironics Actiware V.6.0.9 and R (Core Team 2013) software. Activity data were collected in 30s epochs, the wake threshold was set to low, and the sleep detection interval was set to 20 immobile epochs (i.e., 10 min) for sleep onset and sleep end. Devices were programmed to collect data for 14 consecutive days. The two recordings were scheduled to occur approximately 4–6 weeks apart whenever possible, depending on the health and availability of the participating family (families were instructed to select periods during which their schedules were fairly typical and the individual with Rett‐related syndrome was not experiencing any unusual health issues).

**FIGURE 1:**
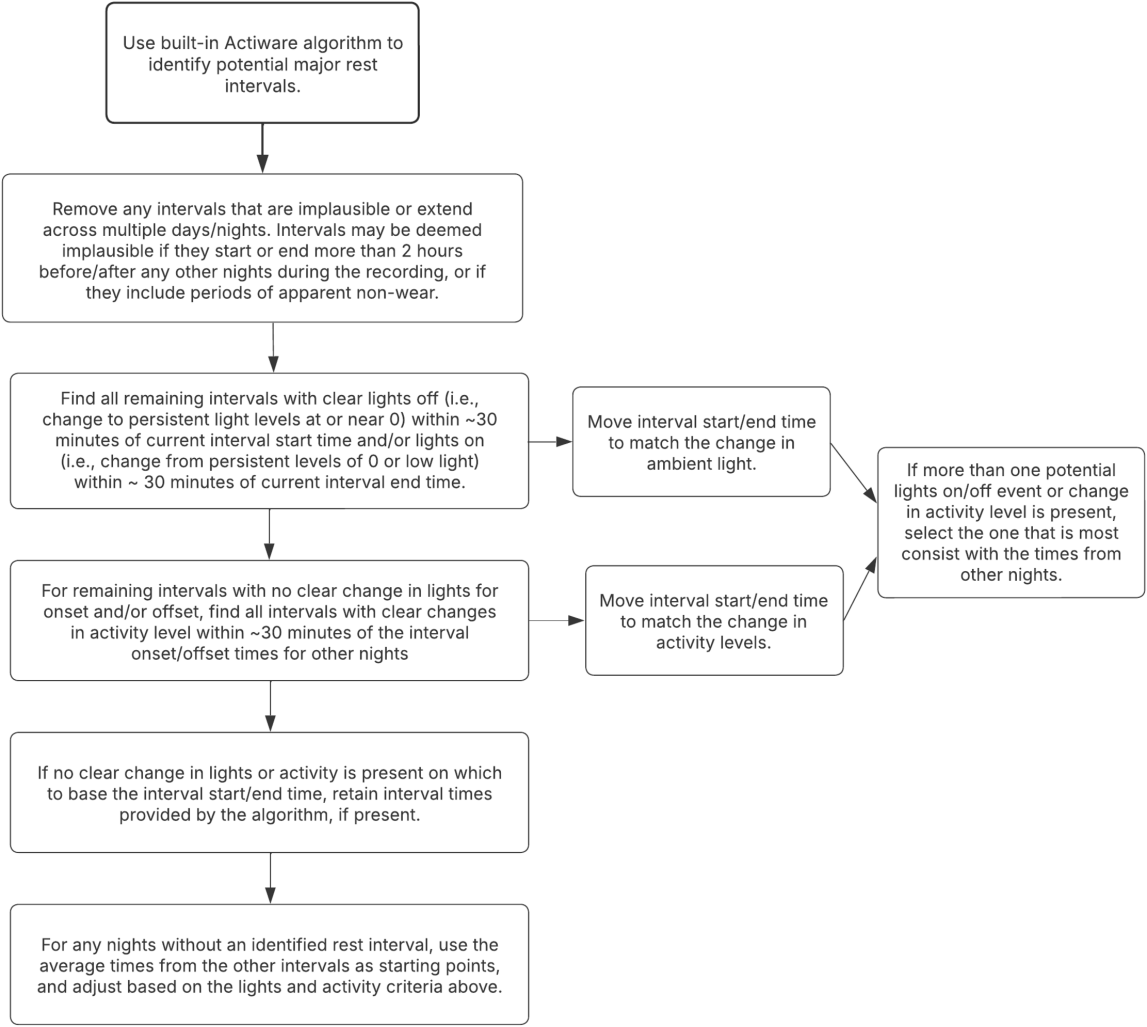
Decision tree for diary‐agnostic algorithm for identifying bed and rise times.

A set of rules was developed to identify bed and rise times without the need to refer to event markers or diaries (i.e., ‘diary agnostic’) that combines the use of the existing Actiware software algorithm for detecting sleep/wake and patterns of light and activity levels (see Figure 1). Although similar sets of rules have been developed for use in other populations (Follesø et al. 2021; Patel et al. 2015), most still include the event markers and diary times as important sources of information. To evaluate the interscorer agreement regarding bed and rise times, a second independent scorer marked the bed and rise times for all nights of a randomly selected subset of the recordings (*n* = 17, 28.3%). Two‐way agreement intraclass correlation coefficients (ICCs) were used to estimate interscorer agreement and agreement between the main scorer and caregiver‐reported event mark and sleep diary times, when available.

For each valid night of recording, several standard sleep quality and quantity metrics were exported directly from the Actiware software from both the sleep and rest interval types calculated by Actiware. Because there is no clear consensus on the specific methods for calculating many sleep quality metrics, particularly regarding appropriate denominators, we applied formulas using the rest interval and sleep interval as the denominator for most sleep measures (see Table 1 for names and descriptions of each variable). Additionally, based on recent evidence documenting the importance of night‐to‐night variability (NNV; Sletten et al. 2023) in sleep duration and timing in health, several NNV metrics were also calculated. For each of these metrics, the root mean square of successive differences (RMSSD) between all of the consecutive nights of the recording was used as an indicator of NNV. For each valid 24‐h period, raw activity counts by epoch were exported to CSV files and analysed for nonparametric circadian parameters using the nparACT package for R (Blume et al. 2016), and cosinor analysis using the circacompare package (Parsons et al. 2020; see Table 2 for names and descriptions of each circadian parameter). To assess the reliability of shorter recording durations, average values were calculated for each recording using the first 3, 5, 7 and 10 nights, as well as for the full 14‐day recording duration.

**TABLE 1:** Sleep duration, quality, and variability metrics and their descriptions.

Variable name	Description
Sleep duration	
Time in bed	Elapsed time between bedtime/lights off to rise time/lights on
Sleep interval duration	Duration of the main nighttime sleep interval
Sleep time	Total number of minutes of sleep during the rest and sleep intervals
Sleep quality and fragmentation	
Sleep onset latency	Elapsed time from bedtime/lights off to beginning of first sleep bout
Sleep percent	Percent of time scored as sleep during the intervals. Calculated for the rest and sleep intervals.
Wake time	Total minutes of wake within the interval. Calculated for the rest and sleep intervals.
Fragmentation	Sum of mobile (above activity threshold) and immobile bouts (below activity threshold) less than 1 min in duration divided by the total number of immobile bouts within the interval. Calculated for the rest and sleep intervals.
Night to night variability (NNV)	Root mean square of successive differences (RMSSD) of total minutes of sleep during the rest interval across consecutive nights
Sleep time NNV	RMSSD of total minutes of sleep during the interval. Calculated for the rest and sleep intervals
Start time NNV	RMSSD of times of start of interval (in decimal hours). Calculated for the rest and sleep intervals.
End time NNV	RMSSD of the end of the interval (in decimal hours). Calculated for the rest and sleep intervals

*Note:* For more information on the calculation of the standard sleep metrics, see the software manual. (Philips Respironics 2015).

Abbreviation: RMSSD = root mean square of successive differences.

**TABLE 2:** Circadian rhythm analysis variables and their descriptions.

Variable name	Description
Cosinor circadian analyses^a^	
MESOR	Midline Estimating Statistic of Rhythm: Rhythm‐adjusted mean of activity counts.
Amplitude	Difference between the MESOR and the maximum height of the average cosinor activity rhythm.
Acrophase	Timing of peak activity levels (in radians).
Nonparametric 24‐h activity metrics^b^	
Interdaily stability (IS)	Stability of rest‐activity rhythms across 24‐h cycles. Varies between 0 and 1, with values closer to 1 indicating greater consistency across days.
Intradaily variability (IV)	Fragmentation of the rest‐activity pattern; frequency and extent of transitions between periods of rest and activity on an hourly basis. Values close to 0 indicate sine‐wave patterns; values approaching 2 indicate Gaussian noise.
L5 activity	Least active 5 h: Average activity counts of the 5 h with the least activity. Should reflect movements during the night, arousals and awakenings.
M10 activity	Most active 10 h: Average activity counts of the 10 consecutive hours with maximal activity. Should reflect daytime activity and may be influenced by daytime napping.
Relative amplitude (RA)	Difference between M10 and L5 activity counts normalised by their sum. Higher values indicate more robust circadian patterns of activity, with higher daytime activity and lower nighttime activity.

^a^ For more information on the cosinor circadian analysis, see (Brown et al. 1990).

^b^ For more information on nonparametric circadian analysis see (Blume et al. 2016) and Gonçalves et al. (Gonçalves et al. 2014).

### 1.3 Data Cleaning and Loss

Periods of nonwear were identified by visual inspection of activity counts to identify extended periods with activity counts of 0 that did not correspond to nighttime sleep periods. All 24‐h periods with more than 10 consecutive missing epochs (i.e., 5 min) during the participant's rest period were excluded from analyses for the standard actigraphic metrics. For analyses of 24‐h activity patterns, periods of missing or invalid data with 120 or fewer consecutive epochs (i.e., 60 mins) were left as 0 s, based on evidence that brief gaps in data have relatively little influence on estimation of circadian parameters (Weed et al. 2022). Files with gaps between 1 and 3 h each were replaced with data imputed using the median time of day method (Weed et al. 2022). Any 24‐h periods with more than 3 h of missing data were excluded from analyses.

### 1.4 Data Analysis

To evaluate test–retest reliability across different recording durations, one‐way ICCs were calculated for each recording duration (i.e., 3, 5, 7, 10 days and full recordings) for each metric using the irr package in R (Gamer and Lemon 2019). Based on existing guidelines for the interpretation of ICCs for assessment of reliability (Fitzpatrick et al. 1998; Streiner et al. 2015), a threshold of 0.70 was used to identify ICCs with adequate evidence of reliability for group‐level decision making, although higher thresholds may be desirable in the context of high‐risk studies, and lower thresholds may be acceptable for low‐risk descriptive studies.

To evaluate the degree to which clinical characteristics may affect the reliability of actigraphy‐derived metrics, exploratory analyses were conducted in which the ICCs were calculated separately for subgroups based on age (i.e., under the age of 12 vs. 12 and older), ambulation status (i.e., walks independently or with assistance vs. does not walk) and seizure status (i.e., no history of seizures or seizures are currently well controlled vs. seizures not controlled). Because the differences in reliability between the metrics based on the rest and sleep intervals were relatively small, overall, these analyses were conducted using only the rest interval data when both were available. Because of the small sample sizes within the subgroups, no statistical comparisons were performed, and results should be interpreted with caution.

## 2 Results

### 2.1 Participant Characteristics and Feasibility

A total of 41 caregivers attempted at least one actigraphy recording session. Recordings from six participants were excluded because of technical problems/equipment failure during one or both recordings: one participant lost the equipment during the collection, one participant did not tolerate wearing the device, one participant was lost to follow‐up, and two participants completed the second recording more than 12 weeks after the first (average time between recordings = 8.0 weeks, minimum = 5.4, maximum = 11.6). Two caregivers reported that their child had experienced a health problem during one of the recordings; because excluding these participants did not result in any changes to the overall pattern of results, they were retained, resulting in 60 separate recordings across 30 participants.

The included participants ranged in age from 2 to 36 years, with a mean of 14.5 years (SD = 9.1) at the time of the first recording. All participants but one were female (*n* = 29, 96.7%). Most, including the male participant, (*n* = 26, 86.7%) had confirmed mutations in the MECP2 gene. Among the 25 females with confirmed MECP2 mutations, 24 had classic or typical Rett syndrome, and 1 had the preserved speech variant. The remaining participants were diagnosed with CDKL5 deficiency disorder (*n* = 1), FOXG1 syndrome (*n* = 1), myocyte enhancer factor 2C (MEF2C) haploinsufficiency disorder with a diagnosis of classic Rett syndrome (*n* = 1) and classic Rett syndrome without genetic testing (*n* = 1). See Table 3 for a full description of participant characteristics.

**TABLE 3:** Participant characteristics.

Clinical characteristic	n	%
Mutation type—MECP2		
Exonic insertion or deletion	3	10.0
p.Arg270*	4	13.3
p.Arg168*	2	6.7
p.Arg133Cys	2	6.7
p.Pro152Arg	2	6.7
C‐terminal mutations	2	6.7
Other frameshift & truncating mutations	7	23.3
Other missense mutations	2	6.7
Intronic variation (male)	1	3.3
Confirmed MECP2 mutation, specifics unknown	1	3.3
Other genetic diagnoses		
CDKL5 deficiency disorder	1	3.3
FoxG1 syndrome	1	3.3
MEF2C mutation	1	3.3
No genetic testing	1	3.3
Age group		
Under 6 years	5	16.7
6–11 years	7	23.3
12–18 years	10	33.3
18 and over	8	26.7
Gross motor function		
Walks without support	11	36.7
Walks with support	4	13.3
Does not walk	15	50.0
Seizure status		
No history of seizures	8	26.7
Previous or well‐controlled with medication	11	36.7
Uncontrolled or poorly controlled	11	36.7
Parent‐reported sleep disturbance		
No disruptive night waking or daytime sleepiness	6	20.0
Occasional night waking or daytime sleepiness	7	23.3
Significant night waking or daytime sleepiness	17	56.7

### 2.2 Data Quality

None of the caregivers reported that the participant shared a bed with another person, meaning that all movement data during the night should be attributable to the target participant. Across the 60 recordings, the number of usable 24‐h periods ranged from 7 to 14 (mean = 13.5, median = 14.0). Specifically, 36 recordings (60.0%) had 14; 22 (36.7%) had 13; 1 (16.7%) had 12; and 1 (16.7%) had 7 days/nights of usable data. Most periods of nonwear occurred at the beginning and end of the 14‐day recording period (i.e., days 1 and 14), with four recordings having one 24‐h period/night excluded in the middle of the recording due to nonwear overnight. Excluding these periods, the number of invalid/missing epochs ranged from 0 to 211 (mean = 6; median = 0). Only one file required imputation for 211 epochs.

Regarding the validity of the cleaning process, ICCs with the human‐scored bedtimes were 0.76 compared with caregiver‐reported times on the sleep diaries, and 0.77 compared with event marker times. Agreement for rise times was slightly lower, but still within the acceptable range (0.72, for diary times; 0.74 for event marker times), suggesting that the cleaning process resulted in comparable bed and rise times to those reported by caregivers when available. Interobserver agreement for bedtimes was very good (ICC = 0.93), and agreement for rise times was somewhat lower, but still well within the acceptable range (ICC = 0.78).

### 2.3 Test–Retest Reliability

ICCs for all average sleep quality, duration and NNV metrics across tested recording durations are reported in Table 4. All average metrics based on rest intervals showed adequate test–retest reliability when averages were based on at least seven nights of recording. Sleep percent (rest interval) and fragmentation (both interval types) required the most nights for reliability, and sleep percent (sleep interval) remained just below the 0.70 cutoff, even at the longest recording durations. Sleep interval duration (time from bed to rise), sleep time (both), sleep onset latency and sleep percent (rest interval) required the fewest nights.

**TABLE 4:** ICCs evaluating test–retest reliability for all average and night‐to‐night variability sleep statistics across increasing recording durations.

Variables	Recording duration				
	3 days	5 days	7 days	10 days	Full rec.
Average sleep statistics					
Time in bed	0.57	0.69	**0.80**	**0.88**	**0.89**
	[0.28, 0.77]	[0.45, 0.84]	**[0.62, 0.90]**	**[0.77, 0.94]**	**[0.78, 0.95]**
Sleep interval duration	0.58	**0.74**	**0.84**	**0.90**	**0.86**
	[0.28, 0.78]	**[0.52, 0.87]**	**[0.69, 0.92]**	**[0.79, 0.95]**	**[0.73, 0.93]**
Sleep time (rest interval)	0.68	**0.75**	**0.82**	**0.90**	**0.91**
	[0.38, 0.84]	**[0.51, 0.87]**	**[0.65, 0.91]**	**[0.80, 0.95]**	**[0.82, 0.96]**
Sleep time (sleep interval)	**0.73**	**0.81**	**0.85**	**0.90**	**0.90**
	**[0.46, 0.87]**	**[0.64, 0.91]**	**[0.70, 0.92]**	**[0.79, 0.95]**	**[0.80, 0.95]**
Sleep onset latency	0.43	**0.82**	**0.85**	**0.90**	**0.83**
	[0.10, 0.68]	**[0.66, 0.91]**	**[0.72, 0.93]**	**[0.80, 0.95]**	**[0.67, 0.91]**
Sleep percent (rest interval)	0.65	**0.78**	**0.81**	**0.87**	**0.89**
	[0.39, 0.82]	**[0.58, 0.89]**	**[0.64, 0.91]**	**[0.75, 0.94]**	**[0.79, 0.95]**
Sleep percent (sleep interval)	0.40	0.36	0.53	**0.73**	**0.76**
	[0.04, 0.66]	[−0.01, 0.63]	[0.22, 0.75]	**[0.51, 0.86]**	**[0.56, 0.88]**
Wake time (rest interval)	0.53	0.66	**0.77**	**0.84**	**0.88**
	[0.23, 0.74]	[0.40, 0.82]	**[0.58, 0.88]**	**[0.70, 0.92]**	**[0.76, 0.94]**
Wake time (sleep interval)	0.26	0.25	0.49	0.69	0.68
	[−0.12, 0.56]	[−0.12, 0.56]	[0.17, 0.72]	[0.44, 0.84]	[0.42, 0.83]
Fragmentation (rest interval)	0.57	0.67	**0.81**	**0.88**	**0.90**
	[0.27, 0.77]	[0.42, 0.83]	**[0.64, 0.91]**	**[0.76, 0.94]**	**[0.80, 0.95]**
Fragmentation (sleep interval)	0.27	0.34	0.66	**0.81**	**0.81**
	[−0.10, 0.57]	[−0.02, 0.62]	[0.40, 0.82]	**[0.63, 0.90]**	**[0.63, 0.90]**
Night to night variability (NNV)					
Sleep time NNV (rest interval)	0.59	**0.70**	**0.76**	**0.89**	**0.89**
	[0.20, 80]	**[0.46, 0.85]**	**[0.55, 0.88]**	**[0.77, 0.94]**	**[0.78, 0.95]**
Sleep time NNV (sleep interval)	0.02	0.53	**0.70**	**0.76**	**0.82**
	[−0.15, 0.51]	[0.22,0.74]	**[0.44, 0.85]**	**[0.55, 0.88]**	**[0.66, 0.91]**
Start time NNV (rest interval)	0.60	**0.70**	0.69	0.55	0.60
	[0.31, 0.79]	**[0.46, 0.85]**	[0.45, 0.84]	[0.24, 0.80]	[0.30, 0.79]
Start time NNV (sleep interval)	0.22	0.43	0.36	0.61	0.53
	[−0.09, 0.51]	[0.10, 0.68]	[0.02, 0.63]	[0.33, 0.79]	[0.22, 0.74]
End time NNV (rest interval)	0.15	0.50	0.62	0.49	0.57
	[−0.23, 0.48]	0.18, 0.72	[0.33, 0.8]	[0.18, 0.72]	[0.28, 0.77]
End time NNV (sleep interval)	0.22	0.04	0.04	0.14	0.57
	[−0.11, 0.52]	[−0.30, 0.38]	[−0.30, 0.38]	[0.20, 0.46]	[0.27, 0.77]

*Note:* ICCs exceeding the 0.70 reliability threshold are reported in bold. Values in brackets are the 95% confidence intervals for each reliability estimate. Full recording was most often 14 days.

For NNV metrics, only sleep time (both interval types) met the 0.70 threshold. Rest interval start time met reliability when only five nights of recording were considered, but this metric was not reliable at other durations, suggesting that it may be particularly sensitive to outliers.

ICCs for all circadian parameters are reported in Table 5. All circadian parameters showed adequate reliability with the full recording duration, but only MESOR, amplitude and M10 activity levels were reliable at the shortest recording durations.

**TABLE 5:** ICCs evaluating test–retest reliability for all circadian parameters across different recording durations.

Variables	Recording duration				
	3 days	5 days	7 days	10 days	Full rec.
Cosinor circadian analyses					
MESOR	**0.90**	**0.89**	**0.91**	**0.94**	**0.93**
	**[0.80, 0.95]**	**[0.77, 0.94]**	**[0.83, 0.96]**	**[0.87, 0.97]**	**[0.87, 0.96]**
Amplitude	**0.79**	**0.78**	**0.83**	**0.87**	**0.89**
	**[0.61, 0.90]**	**[0.58, 0.89]**	**[0.67, 0.91]**	**[0.73, 0.93]**	**[0.80, 0.94]**
Acrophase	0.36	0.41	0.41	0.52	**0.88**
	[−0.01, 0.63]	[0.06, 0.67]	[0.06, 0.67]	[0.21, 0.74]	**[0.75, 0.94]**
Nonparametric circadian analysis					
IS	0.41	0.55	**0.72**	**0.72**	**0.79**
	[0.08, 0.66]	[0.24, 0.75]	**[0.46, 0.86]**	**[0.48, 0.86]**	**[0.61, 0.89]**
IV	0.68	0.66	0.64	**0.71**	**0.74**
	[0.43, 0.83]	[0.40, 0.82]	[0.37, 0.81]	**[0.47, 0.85]**	**[0.55, 0.86]**
RA	0.37	0.49	0.59	0.62	**0.88**
	[0.04, 0.64]	[0.17, 0.71]	[0.30, 0.78]	[0.34, 0.80]	**[0.77, 0.94]**
L5 activity	0.49	0.41	0.57	0.54	**0.73**
	[0.17, 0.72]	[0.08, 0.66]	[0.27, 0.77]	[0.24, 0.75]	**[0.52, 0.85]**
M10 activity	**0.88**	**0.84**	**0.85**	**0.88**	**0.92**
	**[0.70, 0.92]**	**[0.69, 0.92]**	**[0.71, 0.93]**	**[0.75, 0.94]**	**[0.86, 0.96]**

*Note:* ICCs exceeding the 0.70 reliability threshold are reported in bold. Values in brackets are the 95% confidence intervals for each reliability estimate. Full recording was most often 14 days.

### 2.4 Test–Retest Within Clinical Subgroups

The ICCs within each subgroup by age, ambulation status and seizure status are reported in the supplemental materials. Overall, the metrics that showed good reliability in the shortest recording durations in the full group analysis (i.e., MESOR, M10 activity and amplitude) showed relatively consistent reliability coefficients across the various subgroup analyses. Similarly, the metrics that showed relatively poor reliability even at the longest recording durations (i.e., Start time NNV and End time NNV) showed relatively poor reliability across subgroups. For the other metrics, reliability was generally higher among younger vs. older participants, with activity‐acrophase (i.e., timing of daily period with highest activity levels), IV and L5 activity showing the largest differences between the two age groups. Results specific to ambulation status were somewhat more variable, with time in bed, wake time, and amplitude requiring more nights of recording to achieve reliability among those who walk compared to those who do not walk; in contrast, acrophase and L5 activity levels were not reliable at any recording duration among those who do not walk. Differences by seizure status were also inconsistent, with wake time requiring more nights to achieve reliability in those with no or currently controlled seizures relative to those with uncontrolled seizures. In contrast, sleep time was overall more reliable in the no/controlled seizure group relative to the uncontrolled seizure group. Similarly, acrophase and IV were not reliable at any recording duration among the no/controlled seizure group, whereas IS, RA and L5 activity were not reliable at any recording duration among the uncontrolled seizure group. Visual inspection of the raw values suggests that poor reliability in many of the subgroup analyses, particularly for those variables that were more reliable in the full sample, may be attributable to one or two outliers with large differences between recording, the effects of which were exacerbated by the small sample sizes. The specific cases identified as outliers were not consistent across metrics or subgroup analyses, however.

## 3 Discussion

The primary goal of the current study was to identify the number of nights necessary to achieve adequate test–retest reliability for measures of sleep and circadian rhythms in a sample of individuals with Rett and related syndromes. The results suggest that the specific number of days needed to achieve reliability varies according to the specific measures selected. To our knowledge, only two studies have examined the effects of recording duration on the reliability of actigraphy‐derived sleep statistics, with somewhat varying results. Acebo et al. (1999) examined the effects of recording duration on sleep interval duration, total nighttime sleep, wake time and sleep efficiency across different age groups of typically developing children between 12 and 60 months of age. Van Someren (2007) investigated the number of nights needed to achieve reliable estimates of total nighttime sleep and sleep efficiency among a group of adults with insomnia and a group with dementia. For total sleep time, Van Someren found that only one to three nights of recording were necessary to achieve reliability for these two variables, compared to three to seven nights in the Acebo et al. study and five nights in the current sample. For sleep efficiency/sleep percent, Van Someren reported that estimates were reliable with only one to two nights of recording, compared to three to five nights in the Acebo et al. study and five nights in the current analysis. Results across these and the current study are generally consistent in the conclusion that most sleep measures can be reliably estimated when at least seven nights of recording are included.

For the analysis of NNV, we are not aware of any previous work examining the reliability of these measures in any population, although they are used extensively in health outcomes research (see Sletten et al. 2023). Our results suggest that only estimates of variability in total sleep time (using rest or sleep interval) met the reliability threshold. Although variability in bed and rise times has been used in several existing studies, we cannot recommend its use for evaluating night‐to‐night variability in samples of individuals with Rett and related syndromes without additional work investigating the conditions under which these variables are reliable.

We are similarly unaware of any previous work examining the reliability of cosinor circadian analysis metrics. Our results suggest that a full 2‐week recording is needed if the goal is to estimate the acrophase (i.e., timing of peak activity levels) but that average activity levels (i.e., MESOR) and amplitude of the cosine were reliably estimated with only 3 days of recording. In contrast, for the nonparametric circadian parameters only daytime activity levels (i.e., M10 activity) were reliable with very short recording durations. To our knowledge, only van Someren (2007) has reported reliability data for this type of analysis. They reported adequate reliability with one to 2 days for amplitude for individuals with insomnia and those with dementia, which is significantly fewer than in our sample. For interdaily stability (IS), van Someren reported that seven or more days of recording were needed to achieve stability in the dementia group, but estimates were not reliable in the insomnia group for any recording duration (up to a maximum of 7 nights). Our sample met the reliability threshold for IS with approximately 7 days of recording. For intradaily variability (IV), van Someren reported that five or more days of recording were needed to establish adequate reliability, but this increased to six or more days for individuals with insomnia. In our sample, adequate reliability of IV required at least 10 days of recording. Overall, these results suggest that longer recording durations were needed to achieve reliability in the current sample relative to previously reported samples, including those with dementia and insomnia, although these findings should be replicated in additional independent samples.

There are several potential reasons why reliability for some measures was lower in the current sample than has been reported in other populations. One contributing factor is that high fragmentation of activity patterns and increased prevalence of sleep disorders in this population contributed to lower reliability estimates. In addition, our sample included a large proportion of school‐age individuals, and we did not differentiate between weekday and weekend nights, or between periods in which participants were attending school or were at home over school holidays. This may have affected the reliability to some degree, although results from Acebo et al. (1999) suggest that these differences are likely relatively small. Finally, our sample was highly heterogeneous with respect to age and motor ability. Exploratory subgroup analyses suggested that reliability may vary according to these and other clinical characteristics, but the inconsistency in the results and the small sample sizes within each subgroup make interpretation challenging. Furthermore, because the different metrics examined are not independent (e.g., motor ability and seizure frequency typically vary by age in this population), it is unclear based on these analyses the degree to which each individual factor is responsible for any differences in reliability. Furthermore, because we did not have access to medical or genetic records for all participants, we had to rely on the information provided by caregivers for more of the phenotypic and genotypic characterisation, limiting the degree of specificity we could obtain. Additional work in larger samples is needed to further explore how clinical characteristics affect the reliability of actigraphy measures in this and other populations. For all these reasons, the reliability estimates reported in the current analyses should be considered to be sample‐specific, but we believe that they provide a reasonable starting point for researchers considering the use of actigraphy in similar populations.

A challenge in using actigraphy among individuals with Rett and related syndromes (and many other paediatric genetic syndromes and disorders) is that bed and rise times are not self‐reported or self‐determined (i.e., they are put to bed by caregivers), making it impossible to ascertain the time at which the participants intended to go to bed or fall asleep (although such procedures are subject to recall and other forms of bias in all populations). Therefore, bed and rise times are typically imposed on the individual by their caregivers, either based on routine, or on cues that the individual is fatigued/ready to get up for the day. We believe that variables based on rest intervals should be interpreted with caution among individuals who cannot self‐determine their bed/rise times, regardless of their reliability. As noted by Reed and Sacco (2016, 263), the construct of sleep efficiency should be based on the proportion of time the individual is in bed and trying to sleep and as such, should exclude ‘non‐sleep related activities that occur in bed prior to attempting to sleep and those that occur after finally waking,’ but this cannot be determined in our population. Similarly, sleep onset latency should be considered in the context of when the individual is in bed and trying (but failing) to sleep. While the current study shows that latency and sleep efficiency, as defined in the current study, can be estimated reliably, it is unclear whether these measures should be considered comparable to those obtained in populations who can self‐report their bed and wake times.

In collecting data, whether for clinical or research purposes, it is critical to balance the utility of the measures and the burden placed on participants. The data from the current study suggest that researchers should select the number of days of recording based on the reliability of their specific variables of interest at different recording durations. It should be noted, however, that data loss is common in actigraphy studies (e.g., Jang et al. 2020; Ustinov and Lichstein 2013), and this issue may be exacerbated in populations in which caregiver stress is elevated, as well as in the context of repeated or prolonged measurement (e.g., Tonkin et al. 2023). Although our procedure sidestepped the issue of missing data in caregiver‐reported bed and rise times, missing data were still common due to failure to place the device prior to recording time (due to caregiver error in many cases, although delayed courier delivery also occurred). Researchers may therefore choose to ask participants to collect more than the necessary number of nights to ensure enough usable data for their purposes.

Although we believe that the current study provides a useful starting point for those considering using actigraphy to measure sleep and circadian rhythm among individuals with Rett syndrome and other forms of IDD, reliability estimates reported should be considered sample‐specific due to several limitations. The relatively small sample size, although reasonably large for a rare disorder, may have made the reliability estimates more sensitive to outliers. In addition, the sample was not randomly selected or generally representative of any broader population, limiting the generalisability of the findings. Except for the circadian rhythm variables, the current study focused exclusively on nighttime sleep. Most of the caregivers in the current sample reported that the participants frequently took naps during the day, with a few participants showing extreme daytime sleepiness and frequent brief naps throughout the day. Nevertheless, we believe that the focus on nighttime sleep was appropriate for three main reasons: (1) We believe that the quality of nighttime sleep is critical for quality of life for affected individuals and their caregivers; (2) Anecdotally, day‐to‐day variability in daytime sleep was even greater than that of nighttime sleep, which we believe would lead to low overall reliability and (3) Identifying brief periods of daytime sleep is extremely difficult without reliable sleep diary or event markers, particularly among individuals who are highly sedentary or in school or other environments where a caregiver is not present to report naps. For these reasons, we believe that the most reliable, and therefore useful, estimates of sleep will be for nighttime sleep, although the sleep duration estimates do not reflect the total amount of 24‐h sleep for most of the participants in our sample. More research is needed to determine the conditions under which actigraphy can be used in the estimation of daytime and 24‐h sleep patterns among individuals with Rett and related syndromes. Finally, it is important to note that the specific devices and associated software that we used to collect our actigraphy data have been discontinued and are no longer available for purchase. We do not believe that this is a major limitation, however, as multiple studies have shown that many of the actigraphy devices on the market provide comparable data (Jenkins et al. 2022; Lee and Suen 2017).

In conclusion, the current study provides evidence that most sleep and circadian rhythm parameters extracted from actigraphy data can be reliably estimated among individuals with Rett and related syndromes, although the minimum number of nights varies considerably based on the specific parameters selected. The results also indicate that caregiver‐reported diaries and event markers may not be necessary to obtain stable estimates. Nevertheless, additional work is needed to determine which statistics show evidence of current or predictive validity before they can be recommended as potential outcome measures for use in clinical trials and other clinical research in this and similar populations.

## Funding

This study was supported, in part, by funding from the National Institutes of Child Health and Human Development (Grant # R21HD101075).

## Conflicts of Interest

The authors declare no conflicts of interest.

## Supporting information


**Data S1:** Supporting Information.

## Data Availability

Because the data were collected from individuals with rare genetic disorders, some of the data may be identifiable. Data files are made available upon reasonable request.
